# Understanding the involvement of HtrA4 during syncytiotrophoblast differentiation

**DOI:** 10.3389/fcell.2026.1926149

**Published:** 2026-09-29

**Authors:** Bothidah Thach, Yao Wang, Chaitali Dekiwadia, Guiying Nie

**Affiliations:** 1 Implantation and Pregnancy Laboratory, School of Health and Biomedical Sciences, RMIT University, Bundoora, VIC, Australia; 2 RMIT Microscopy and Microanalysis Facility, STEM College, RMIT University, Melbourne, VIC, Australia

**Keywords:** cell fusion, HtrA4, placenta, syncytialization, syncytiotrophoblast, trophoblast, trophoblast differentiation, trophoblast stem cells (TSCs)

## Abstract

The syncytiotrophoblast (STB), the outermost layer of the human placenta, is vital to pregnancy health as it functions as the direct maternal-fetal interface. Being terminally differentiated and multinucleated, STB cannot divide but is formed/sustained by fusion of cytotrophoblasts throughout pregnancy. HtrA4, produced only in placentas of humans and high-order primates, is obligatory for syncytialization, including differentiation of trophoblast stem cells (TSCs) into STB as HtrA4 knockdown (KD) blocks the process; however, how HtrA4 mediates the process remains unknown. The current study aimed to understand how HtrA4 is involved in STB differentiation, by analyzing HtrA4-KD TSCs using mRNA transcriptomics and scanning electron microscopy (SEM). We first investigated how control and HtrA4-KD TSCs, respectively, altered protein-coding gene expressions globally upon stimulation for STB differentiation, then compared the significantly differentially expressed genes (DEGs) to identify the deficiencies of HtrA4-KD cells. Among the DEGs associated with STB differentiation of controls, 25% of them also occurred in HtrA4-KD cells, implying these were HtrA4-independent; in contrast, the remaining 75% DEGs were severely attenuated or completely blocked in HtrA4-KD cells, signifying these were HtrA4-modulated or HtrA4-dependent. Analysis of biological processes, molecular functions, and cellular pathways associated with these distinctive DEGs suggested that early STB differentiation events, such as ceasing cell division and initiating autophagy, were un-influenced by HtrA4. However, many subsequent processes of STB differentiation were partially or fully influenced by HtrA4, some of which included hormone activity, cell surface and extracellular matrix remodeling, adjustments in cell-cell signaling, and restructuring of various components of the cytoplasm/cytoskeleton. Moreover, our data suggested that pathways related to nervous system development were suppressed during STB differentiation, and this was influenced by HtrA4. Furthermore, it was indicated that altering protein binding was a major juncture of HtrA4 influence, and mitigating oxidative stress during STB differentiation might be tightly coupled with HtrA4. By SEM examination of the morphological changes along the time course of STB differentiation, we further visually demonstrated that HtrA4-KD cells commenced the differentiation process, but were blocked from progressing into multinucleated STB. Collectively, these findings provide important new insights into the pivotal role of HtrA4 in STB differentiation.

## Introduction

The placenta is a transient organ of fetal origin that forms only during pregnancy; it is essential for supporting the growth and development of the conceptus throughout gestation (Turco and Moffett, 2019). Key functions of the placenta are contingent on specialized epithelial cells termed trophoblasts, which include three main types: cytotrophoblasts (CTBs), extravillous trophoblasts (EVTs), and the syncytiotrophoblast (STB). The STB covers the surface of all placental villi, forming a vital aspect of the maternal-fetal interface. This layer houses numerous nuclei within a single continuous cytoplasm and possesses no intercellular borders (Renaud, 2026). The unique morphology of this specialized giant cell enables the efficient transfer of nutrients, waste, and gases between the maternal and fetal systems, while also acting as a physical and immunological barrier to protect the fetus (Yong et al., 2021). Moreover, the STB performs an important endocrine role via the synthesis and release of placental factors and hormones to facilitate maternal-fetal communication (Yong et al., 2021). Thus, the development and maintenance of STB is crucial for placental function and pregnancy outcomes.

One major characteristic of the STB is that it is terminally differentiated and cannot divide to grow or repair; instead, the syncytial layer is continuously replenished and renewed by the fusion of underlying villous CTBs through a process known as syncytialization (Renaud, 2026). The fusion of CTBs requires a dramatic shift in plasma membrane and cytoskeletal dynamics as these mononuclear cells transform and incorporate into the syncytial layer (Renaud and Jeyarajah, 2022). These changes include exit from cell cycle (Crocker et al., 2007), loss of junctional proteins (Pidoux et al., 2014; Pidoux et al., 2010), and extensive membrane remodeling (Moore et al., 2021). Aberrant or incomplete differentiation of CTBs is implicated in placental dysfunction and pregnancy complications including preeclampsia, placenta accreta syndrome, and intrauterine growth restriction (Wang M. et al., 2024; Benouda et al., 2025; Chen et al., 2025).

High temperature requirement factor A4 (HtrA4) is a serine protease that is produced only in the placenta of humans and a few high order primates (Singh et al., 2015; Wang Y. et al., 2024). Within the human placenta, HtrA4 is limited to trophoblasts, localizing to CTBs, EVTs and STB (Inagaki et al., 2012). Our recent studies strongly suggest that HtrA4 plays a critical role in trophoblast differentiation into STB as well as EVTs (Wang Y. et al., 2024; Thach et al., 2024), and its importance in STB differentiation has been illustrated in several systems. HtrA4 is dramatically upregulated when primary CTBs of term placenta spontaneously syncytialize in culture (Mansilla et al., 2021). Human choriocarcinoma BeWo cells also significantly upregulate HtrA4 expression during fusion, and these cells fail to syncytialize when HtrA4 is knocked out (Mansilla et al., 2021; Pei et al., 2023). We have further shown that HtrA4 is likewise critical for CTB fusion in cultured human placental explants (Mansilla et al., 2025). Moreover, we have isolated human trophoblast stem cells (TSCs) from first trimester placentas, and reported that these TSCs also intensely increase HtrA4 expression when differentiating into multinucleated STB, up-regulating well-known syncytial markers such as human chorionic gonadotropin (hCG) and syncytin-1 and down-regulating E-cadherin (Thach et al., 2024). Using CRISPR/Cas9-mediated gene silencing technologies, we have created stable HtrA4-knockdown (HtrA4-KD) TSCs, and showed that these cells fail to increase HtrA4 production upon stimulation for STB differentiation, and remain primarily mononucleated with no significant STB differentiation (Thach et al., 2024). Together, these results have demonstrated that HtrA4 plays a critical role in enabling the differentiation of TSCs into STB.

The STB is responsible for placental secretion, including that of HtrA4 which is released into maternal circulation throughout pregnancy (Singh et al., 2015; Inagaki et al., 2012). Circulating levels of HtrA4 are aberrantly elevated in the pregnancy complication preeclampsia, especially in the early-onset subtype (Singh et al., 2015; Wang and Nie, 2016; Wang et al., 2019a; Wang et al., 2019b; Yuan et al., 2023). Higher levels of HtrA4 present in maternal circulation likely contribute to endothelial dysfunction and the development of preeclampsia (Wang and Nie, 2016; Wang et al., 2019a; Wang et al., 2019b; Tseng et al., 2019; Wang et al., 2020). While these studies highlight the potential pathological role of HtrA4 elevation in maternal circulation, the fundamental functions of HtrA4 in normal placental development and function require further investigation.

The current study aimed to understand how HtrA4 is involved in STB differentiation, and whether it is required for its initial commencement or subsequent completion. Utilizing the stable HtrA4-KD TSCs and control TSCs that we established previously (Thach et al., 2024), and applying global mRNA transcriptomics, the study first examined how control and HtrA4-KD TSCs respectively altered protein-coding gene expression upon stimulation for STB differentiation. Following validation by RT-PCR and immunofluorescence, mRNA changes in control and HtrA4-KD cells were compared to find similarities and differences, thereby identifying transcriptomic impairment of HtrA4-KD cells during STB differentiation. Furthermore, these gene changes were bioinformatically analyzed to determine their associated biological processes, molecular functions, cellular components and pathways, thereby deducing which major STB differentiation processes were HtrA4 dependent or independent. Scanning electron microscopy (SEM) was then applied to scrutinize the topography of control and HtrA4-KD cells along the time course of STB differentiation, further investigating where HtrA4 suppression impeded the fusion process.

## Materials and methods

### Culture of TSCs and differentiation into STB

The current investigation utilized stable HtrA4-KD TSCs and control TSCs created in our previous study (Thach et al., 2024). In short, TSCs were isolated from first trimester placental villi by enzymatic digestion and immuno-purification (Thach et al., 2024), and their identity was confirmed by the presentation of the epithelial marker CK7 and the stem cell marker CD49f, alongside the absence of the fibroblast marker vimentin, as previously published (Thach et al., 2024; Okae et al., 2018). The knockdown of HtrA4 gene expression was achieved by CRISPR/Cas9-mediated gene silencing, control cells were similarly transfected with an empty vector (Thach et al., 2024). Stable HtrA4 knockdown was validated by real-time RT-PCR, immunofluorescence, and ELISA in HtrA4-KD TSCs with and without stimulation for STB differentiation (Thach et al., 2024).

These HtrA4-KD and control TSCs were cultured in this study as formerly reported (Thach et al., 2024). In brief, TSCs were maintained at 37 °C under 5% CO_2_, with culture media replaced every 2–3 days. For transcriptomic and real-time RT-PCR analyses, TSCs were seeded onto 12-well plates pre-coated with collagen IV (Sigma-Aldrich). Cells were cultured for 6 days in TSC media (as un-stimulated controls) or in STB media (to induce differentiation). Media were replenished on day 3, and cells were harvested on day 6 for RNA isolation. The primary difference between these media formulations was that the TSC media contained stemness maintenance factors, including CHIR-99021 and SB431542 (both from Selleckchem, TX, USA), A83-01 (Tocris Bioscience, Bristol, United Kingdom), L-ascorbic acid and VPA (Sigma-Aldrich), EGF (PeproTech, NJ, USA) and Y27632 (STEMCELL), whereas STB media omitted these factors but contained KnockOut serum Replacement (KSR, Thermo Fisher Scientific), forskolin (2 μM, Sigma-Aldrich), lower levels of Y27632, and removal of FBS (Thermo Fisher Scientific) to drive differentiation (Thach et al., 2024). For immunofluorescence studies, TSCs were seeded onto 10 mm glass coverslips (Marienfeld, Lauda-Königshofen, Germany) pre-coated with collagen IV and placed into 48-well plates, cultured for 6 days in TSC or STB media with media replenishment also on day 3, and collected on day 6 (Thach et al., 2024). For SEM studies, TSCs were plated onto collagen IV-coated 10 mm glass coverslips and cultured in 48-well plates in TSC or STB media as described above, but harvested on days 0, 2, 3, 4 and 6 along the time course of STB differentiation. Culture media from this experiment were also collected for further analysis.

### RNA extraction, mRNA-seq and bioinformatic analysis

Total RNA was extracted using TRI Reagent (Sigma-Aldrich) and treated with a DNA-free kit (Invitrogen) in accordance with the manufacturers’ protocols. RNA quality and concentration were assessed by Nanodrop ND-1000 (Thermo Fisher Scientific) as previously described (Thach et al., 2024). Total RNA (n = 3 from each cell type and each treatment) was sent to GenXPro GmbH (Frankfurt am Main, Germany) for mRNA-seq, using their patented method of Massive Analyses of cDNA Ends (MACE-Seq) and Unique Molecular Identifiers (Pat. Nr. 102008025656) at a sequencing depth of 10 million reads. In brief, samples underwent quality control by LabChipDXII and Qbit, then 100 ng RNA was used for library preparation with an in-house Rapid MACE-Seq kit (GenXPro GmbH, Germany). Fragmented RNA underwent reverse transcription using barcoded oligo (dT) primers containing TrueQuant unique molecular identifiers, followed by template switching. PCR-amplified libraries were purified using solid phase reversible immobilization beads, then sequenced on the Illumina platform NextSeq 500. Unprocessed sequencing reads were adapter- and quality-trimmed using Cutadapt (version 4.6) (Martin, 2011), deduplication (based on Unique Molecular Identifier) was performed using in-house tools, and read quality was assessed with FastQC (version 0.11.9) (Andrews, 2010). Processed reads were mapped with a reference genome using Bowtie2 (version 2.4.4) (Langmead and Salzberg, 2012), or nvBowtie from NVBIO (Pantaleoni and Subtil, 2015), and quantified using HTSeq (version 2.0.2) (Anders et al., 2015). Raw counts were geometric-mean-normalized using DESeq2 (version 1.38) (Love et al., 2014), then TPM-normalized (dividing each count with the sum of all counts for each sample multiplied by one million). Quantification of mapped reads to each transcript was performed using HTSeq (version 2.0.2) (Anders et al., 2015), and differential gene expression analysis was carried out using DESeq2 (version 1.38) (Love et al., 2014).

We analyzed the dataset generated above to firstly identify significantly differentially expressed genes (DEGs) in control TSCs and HtrA4-KD TSCs, respectively, upon stimulation for STB differentiation, using the inclusion criteria of protein-coding genes only, P ≤ 0.05, fdr (false discovery rate) ≤ 0.1, and fold change (fc) ≥ 2. These respective DEGs were visualized with heatmaps generated in RStudio (Version 4.4.1, Posit Software, PBC, Boston, MA, USA) using the heatmap.2 function of the gplots package. To understand how HtrA4-KD cells fail to fully differentiate into STB, we next compared the above DEGs of control and HtrA4-KD cells to identify which DEGs were shared between the 2 cell types upon stimulation for STB formation, and which DEGs occurred uniquely, especially DEGs that appeared in control cells but were absent in HtrA4-KD cells. These different groups of DEGs were then analyzed using the online bioinformatic tool DAVID (Huang da et al., 2009a; Huang da et al., 2009b) to identify enriched biological terms and over-represented pathways within the online pathway knowledgebases of Gene Ontology (GO) (Ashburner et al., 2000; Gene Ontology et al., 2023) and KEGG (Kanehisa, 2019; Kanehisa et al., 2025; Kanehisa and Goto, 2000). The top 15 enriched terms for each DEG group were identified based on significance (-log_10_ [P-value], the larger the more significant) in conjunction with gene count (a minimum of 5 genes per term), and plotted using the ggplot package in RStudio. Finally, interaction maps between relevant enriched terms were generated using Cytoscape (Version 3.10.3, Cytoscape Consortium).

### Real-time RT-PCR

Complementary DNA (cDNA) was synthesized using 450 ng RNA with SuperScript III First-Strand kit (Thermo Fisher Scientific). Real-time RT-PCR analysis was performed on a QuantStudio™ 7 Flex Real-Time PCR System (Thermo Fisher Scientific) using the following conditions: 1) 95 °C for 10 min; 2) 40 cycles of denaturation (15 s at 95 °C), annealing and extension (40 s at 60 °C); and 3) a dissociation curve assessment between 60 °C and 95 °C with continuous fluorescence measurement. PCR reactions were prepared to a final 10 μL with 4 μL cDNA (diluted 1:80), 5 μL SYBR Green PCR Master Mix (Thermo Fisher Scientific) and 1 μL primers (1 μM final concentration). Primer details are listed in Table 1. Gene expression levels were normalized to 18 S rRNA (which remained stable across TSC and STB groups) and expressed as fold change using the ΔΔCt method. All samples were run in triplicate, and each experiment was independently repeated 4 times.

**TABLE 1:** Primers used for RT-PCR analysis.

Gene/Product size	Forward	Reverse
*ADAM12* 203 bp	5′-CTT​ATG​GCA​ACT​GTG​GCA​AA-3′	5′-GTC​ATC​GCC​CAA​GTA​CAC​G-3′
*BPGM* 117 bp	5′-ATC​AGA​AAC​TCA​ACA​GCG​AAG​G-3′	5′-TGT​GAA​TGG​ACC​GAT​TAA​GGA​C-3′
*CAPN6* 221 bp	5′-ACT​ATG​GGT​CCT​CCT​CTG-3′	5′-AGC​TGG​TGG​TTG​CTA​ATG-3′
*COMT* 203 bp	5′-CGA​CTG​TGC​CGC​CAT​CA-3′	5′-GCA​GCA​GGC​CAC​ATT​CCT​C-3′
*ENG* 95 bp	5′-CAT​CCT​TGA​AGT​CCA​TGT​CCT​CTT-3′	5′-GCC​AGG​TGC​CAT​TTT​GCT​T-3′
*FSCN1* 500 bp	5′-TCC​CGA​CTG​CCG​TTT​CCT-3′	5′-GCT​CGT​CCT​TGC​CCA​CCT​T-3′
*FURIN* 73 bp	5′-GAG​ATT​GAA​AAC​ACC​AGC​GAA-3′	5′-CGG​TGC​CAT​AGA​GTA​CGA​G-3′
*MKI67* 188 bp	5′-TTG​GTA​CTG​GGG​GAG​GGA​GA-3′	5′-TGG​GAG​GCG​AAA​AAG​TAA​AA-3′
*NECTIN1* 213 bp	5′-TAC​ATC​TGC​GAG​TTT​GCT​AC-3′	5′-CTC​ACC​TTT​TAA​CCG​AGT​TTT-3′
*PEG10* 110 bp	5′-GGA​CCT​GGA​TTG​GAA​CGA​G-3′	5′-GAG​CAG​ACA​GCG​ACT​TGG-3′
*STMN1* 173 bp	5′-AAG​ACG​CAA​GTC​CCA​TGA​AG-3′	5′-CCA​TTT​GTG​CCT​CTC​GGT​T-3′
*TEAD4* 182 bp	5′-CAG​TAT​GAG​AGC​CCC​GAG​AA-3′	5′-TGC​TTG​AGC​TTG​TGG​ATG​AA-3′
*18s* 187 bp	5′-CGG​CTA​CCA​CAT​CCA​AGG​AA-3′	5′-GCT​GGA​ATT​ACC​GCG​GCT-3′

bp = base pairs.

### Immunofluorescence

Immunofluorescence was performed as previously described (Thach et al., 2024). All procedures were carried out at room temperature unless stated otherwise. Cells were fixed with 4% paraformaldehyde (VWR, PA, USA) for 10 min, then permeabilized with 0.1% Triton X-100 (Sigma-Aldrich) in PBS for 5 min. They were next blocked with 1% BSA in PBS for 2 h, then incubated at 4 °C overnight with antibodies against TEAD4 (mouse monoclonal, 5 μg/mL, H00007004-M01A, Abnova, Jhongli, Taiwan), or CD105 (also called endoglin, ENG) (rabbit monoclonal, 12 μg/mL, MA5-35197, Invitrogen), or furin (rabbit polyclonal, 2 μg/mL, sc 20,801, Santa Cruz Biotechnology, Dallas, TX, USA). IgGs derived from the mouse (Invitrogen) or rabbit (Dako) were included as negative controls. Steps from here onwards were performed in the dark. Cells were incubated for 2 h with either a goat anti-mouse Alexa Fluor 568 antibody or a donkey anti-rabbit Alexa Fluor 568 antibody (all diluted 1:200, Thermo Fisher Scientific). Nuclei were then counter-stained with DAPI (4′,6-Diamidino-2-phenylindole dihydrochloride, 1:2000 dilution, Sigma-Aldrich) for 10 min, and coverslips were mounted with fluorescent mounting media (Dako). Staining was visualized using an Olympus BX60 fluorescent microscope (Olympus, Shinjuku, Tokyo, Japan). Images were taken with a Nikon DS-Ri2 camera (Nikon, Minato City, Tokyo, Japan) and analyzed using Olympus CellSens software. Three to four random images per condition were taken, and experiments were independently repeated three times.

### Scanning electron microscopy (SEM)

Cells were cultured and treated on coverslips as described above, then collected and processed as follows, with all washing steps performed at room temperature on a plate rocker. Cells were first rinsed with DMEM/F-12+GlutaMAX™ serum-free media (Thermo Fisher Scientific) for 1 min, washed with PBS for 3 min, then fixed in 4% paraformaldehyde (prepared in PBS) for 30 min. These cells were then rinsed with PBS three times (1 min each) and washed with MilliQ water twice (5 min and 3 min). Next, they were dehydrated with increasing concentrations of ethanol in MilliQ water (50% for 10 min, 90% for 10 min, 100% for 15 min and repeated), then placed in 100% ethanol for at least 30 min. Coverslips were then mounted onto metal specimen mounts using carbon tape and air-dried in a fume hood for at least 1 h. Finally, samples were coated with gold palladium (10 nm thickness) with a Leica ACE600 High Vacuum Sputter Coater (Leica Microsystems, Wetzlar, Germany), and imaged using a FEI Quanta 200 Scanning Electron Microscope (Thermo Fisher Scientific) under high vacuum conditions, with accelerating voltage of 3.0 kV, spot size of 5 and working distance of 10 mm.

SEM images were analyzed to quantify visible changes in cell morphology, including features of nuclei and nucleoli. All measurements and counts were performed using ImageJ (v1.54g) (S et al., 2012). Cell fusion was also quantified as previously reported (Mansilla et al., 2021; Omata et al., 2013). Briefly, the number of nuclei within each cell (with two or more nuclei in multinucleated cells) were first counted per image, then the fusion index was calculated as the percentage of nuclei present within multinucleated cells relative to the total number of nuclei. The number of nucleoli per nucleus was also counted. Data from three images were averaged for each sample, and each experiment was independently repeated three times.

### Enzyme-linked immunosorbent assay (ELISA)

Secreted HtrA4 was measured in the culture media collected from the SEM experiment. These media were collected after 0, 2, 3, 4 and 6 days of culture; day 6 culture underwent media replenishment on day 3. ELISA was performed as previously described (Thach et al., 2024). In brief, half-area high binding Corning 96-well microplates (Sigma-Aldrich, USA) were coated with the coating antibody overnight at 4 °C, blocked with 1% (w/v) BSA (Sigma–Aldrich, USA) in PBS for 90 min at 37 °C, then incubated with culture media overnight at 4 °C. Plates were then incubated sequentially with the biotinylated detection antibody for 1 h at 37 °C, streptavidin-HRP (Dako, USA) for 50 min at room temperature, and 1-Step Ultra TMB (Thermo Fisher Scientific, USA) for 15 min at room temperature. Sulfuric acid was then added to each well, and the absorbance at 450 nm was determined on a plate reader (Clariostar, BMG Labtech, Australia). Between-incubation washes were carried out with PBS containing 0.05% (v/v) Tween using an automated plate washer (ELx50 Washer, BioTek, USA).

### Statistical analysis

Statistical analyses were performed using GraphPad PRISM (Version 10.3.1, GraphPad Software Inc., CA, USA). Unpaired t-tests were used to compare parameters of TSCs with and without stimulation for STB differentiation. Two-way ANOVA with Bonferroni’s multiple comparisons was employed to compare control and HtrA4-KD cells at each timepoint for various factors associated with SEM analyses. Data were expressed as mean ± SD. Statistical values for bioinformatic analyses were generated by DAVID. Statistical significance was defined as *P ≤ 0.05, **P ≤ 0.01, ***P ≤ 0.001, ****P ≤ 0.0001.

## Results

### Identification of DEGs in control and HtrA4-KD TSCs upon stimulation for STB differentiation

We first investigated how control and HtrA4-KD TSCs altered expression of protein-coding genes upon stimulation for STB differentiation. Control and HtrA4-KD TSCs were cultured without and with stimulation for STB differentiation for 6 days; their total RNA was then analyzed by RNA-seq (n = 3 per group per condition), which identified a total of around 19,900 protein-coding transcripts. Next, mRNAs that significantly differed in levels upon stimulation for STB differentiation in control and HtrA4-KD cells, respectively, were identified using the criteria of P-value ≤0.05, fdr ≤0.1, and fc ≥ 2. Control cells showed a total of 2,352 DEGs, with 1,289 being upregulated and 1,063 downregulated for STB differentiation (Figure 1A). In contrast, a total of 1373 DEGS were identified in HtrA4-KD cells, including 729 upregulated and 644 downregulated (Figure 1A). These results indicate that significant mRNA changes occurred in both control and HtrA4-KD cells upon stimulation for STB differentiation, but the extent of changes was considerably attenuated in HtrA4-KD cells.

**FIGURE 1:**
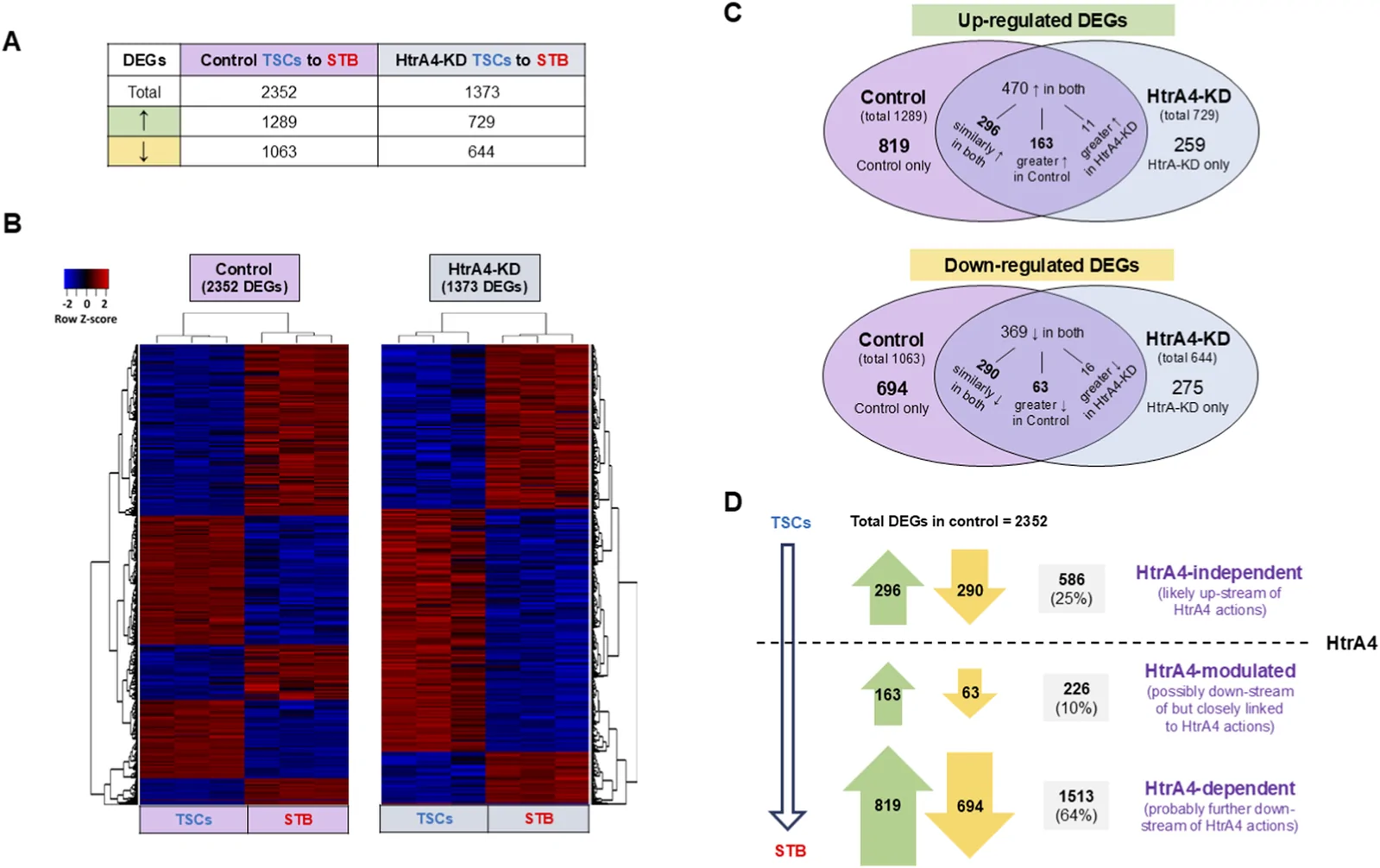
Identification and comparison of differentially expressed genes (DEGs) in control and HtrA4-KD TSCs upon stimulation for STB differentiation. **(A)** Summary of significant DEGs upregulated (↑) or downregulated (↓) in control and HtrA4-KD TSCs respectively following stimulation for STB differentiation (P ≤ 0.05, fc ≥ 2, fdr ≤0.1). **(B)** Heatmap of DEGs shown in **(A)** for control and HtrA4-KD cells, respectively. Normalized gene expression values were standardized by row Z-score and hierarchical clustering was visualized by row and column dendrograms (n = 3). The heatmap color range denotes lowest values in blue and highest in red. **(C)** Venn diagrams showing the number of upregulated (↑) and downregulated (↓) DEGs in control and HtrA4-KD cells, respectively, indicating whether they were observed in both cell types or only in one. DEGs seen in both cell types were further separated into three groups, as shown in the intersection. **(D)** Grouping of DEGs found in control cells shown in **(A)** into three main categories (↑ and ↓ for each): HtrA4-independent (observed similarly in both control and HtrA4-KD cells, thus likely up-stream of HtrA4 actions), HtrA4-modulated (detected in both but to a greater magnitude in control than HtrA4-KD cells, thus likely down-stream of but closely linked to HtrA4 actions), and HtrA4-dependent (detected in control but not in HtrA4-KD cells, thus likely further down-stream of HtrA4 actions).

The above DEGs were further analyzed using heatmaps for control and HtrA4-KD cells respectively, by plotting their expression levels in un-stimulated (TSCs) and stimulated (STB) cells (Figure 1B). Normalized gene expression values were standardized by row Z-score, with levels represented on a color scale (blue indicating lower and red higher levels). Each row represented a distinctive gene and each column an individual sample, which were ordered automatically by hierarchical clustering based on degree of similarity and visualized by dendrograms. In both control and HtrA4-KD groups, TSC and STB samples clustered separately as expected, demonstrating considerable differences between the 2 cell states (Figure 1B).

### Comparative analysis of control and HtrA4-KD DEGs identifies diverse DEG sub-groups

Our previous studies have indicated that control TSCs undergo successful STB differentiation, whereas HtrA4-KD TSCs are incapable of full differentiation (Thach et al., 2024). To understand how HtrA4-KD cells are blocked from full differentiation, we next compared the DEGs identified in both cell types following STB stimulation to identify shared and unique DEGs. For this analysis, upregulated and downregulated DEGs were evaluated separately.

For the upregulated DEGs (a total of 1,289 in control and 729 in HtrA4-KD cells), 470 were found in both cell types, whereas 819 were observed exclusively in control and 259 uniquely in HtrA4-KD (Figure 1C). Further analysis of the common 470 DEGs revealed that while 296 of them were similarly upregulated in both cell types, the magnitude of upregulation for 163 was greater in control cells, whereas that for the remaining 11 was greater in HtrA4-KD cells (Figure 1C).

For the downregulated DEGs (a total of 1,063 in control and 644 in HtrA4-KD cells), 369 were reduced in both cell types, while 694 were downregulated uniquely in controls, and 275 were lowered exclusively in HtrA4-KD cells (Figure 1C). When the 369 commonly downregulated DEGs were further analyzed, 290 were found to be similarly reduced in both cell types, whereas the remaining 63 were downregulated to a greater extent in control cells, and 16 reduced more severely in HtrA4-KD cells (Figure 1C).

These similarities and differences suggest that while some differentiation processes did occur in HtrA4-KD cells upon stimulation, not all changes displayed by the control cells arose in HtrA4-KD cells. To identify the shortcomings of HtrA4-KD cells in STB differentiation, we reasoned that all DEGs presented in control cells represented genuine mRNA changes associated with or required for the differentiation, whereas DEGs that occurred uniquely or more intensely in HtrA4-KD cells likely resulted from reactive or compensatory responses to the lack of HtrA4 rather than authentic STB differentiation. Based on this rationale, we next focused on the DEGs identified in control cells and separated them into the following sub-groups (Figure 1D): those that were also similarly up- or downregulated in HtrA4-KD cells (296 and 290, respectively); those that increased or decreased to a greater extent in control than HtrA4-KD cells (163 and 63, respectively); and those that rose or declined exclusively in controls but not in HtrA4-KD cells (819 and 694, respectively).

DEGs that occurred similarly in both control and HtrA4-KD cells, 586 in total (296 up- and 290 downregulated), representing 25% of all DEGs, were then categorized as HtrA4-independent (Figure 1D); these were tentatively speculated to likely occur up-stream of HtrA4 actions (Figure 1D). DEGs that changed to a greater degree in control than in HtrA4-KD cells, 226 in total (163 up- and 63 downregulated), comprising 10% of total DEGs, were postulated to be HtrA4-modulated, and potentially positioned down-stream of but closely linked to HtrA4 actions (Figure 1D). DEGs observed only in controls but completely blocked in HtrA4-KD cells, a total of 1,513 (819 up- and 694 downregulated), constituting 64% of all DEGs, were classified as HtrA4-dependent, and posited to probably lie further down-stream of HtrA4 actions (Figure 1D). Consequently, these DEGs associated with authentic STB differentiation were separated into six sub-groups: up- or downregulated and HtrA4-independent (25% of total control DEGs), up- or downregulated and HtrA4-modulated (10% of DEGs), and up- or downregulated and HtrA4-dependent (64% of DEGs). These distinctive groups of DEGs were subsequently analyzed as the genuine constituents of STB differentiation to understand how HtrA4 is involved in the differentiation process.

For DEGs that occurred uniquely in HtrA4-KD (259 up- and 275 downregulated) or changed more profoundly in HtrA4-KD than in controls (11 up- and 16 downregulated, accounting for 1% of control DEGs), as shown in Figure 1C, these changes likely reflected cellular responses to HtrA4 deficiency rather than authentic STB differentiation. Consequently, these DEGs were analyzed separately to complement our understanding of the role of HtrA4 in STB differentiation.

### Validation of representative DEGs by real-time RT-PCR

We next validated the RNAseq data by real-time RT-PCR analysis. Two representative DEGs from each of the above six sub-categories were analyzed in control and HtrA4-KD cells without and with stimulation for STB differentiation (Figure 2). For upregulated HtrA4-independent DEGs, transcripts of bisphosphoglycerate mutase (*BPGM*) and calcium-dependent cysteine protease Calpain 6 (*CAPN6*) were examined (Figure 2A); both significantly increased upon stimulation for STB differentiation in both cell types (Figure 2A). For downregulated HtrA4-independent DEGs, transcriptional enhancer TEA Domain Transcription Factor 4 (*TEAD4*) and marker of proliferation Ki-67 (*MKI67*) were assessed (Figure 2B); again, both showed a marked decrease upon stimulation for STB differentiation in both cell types (Figure 2B).

**FIGURE 2:**
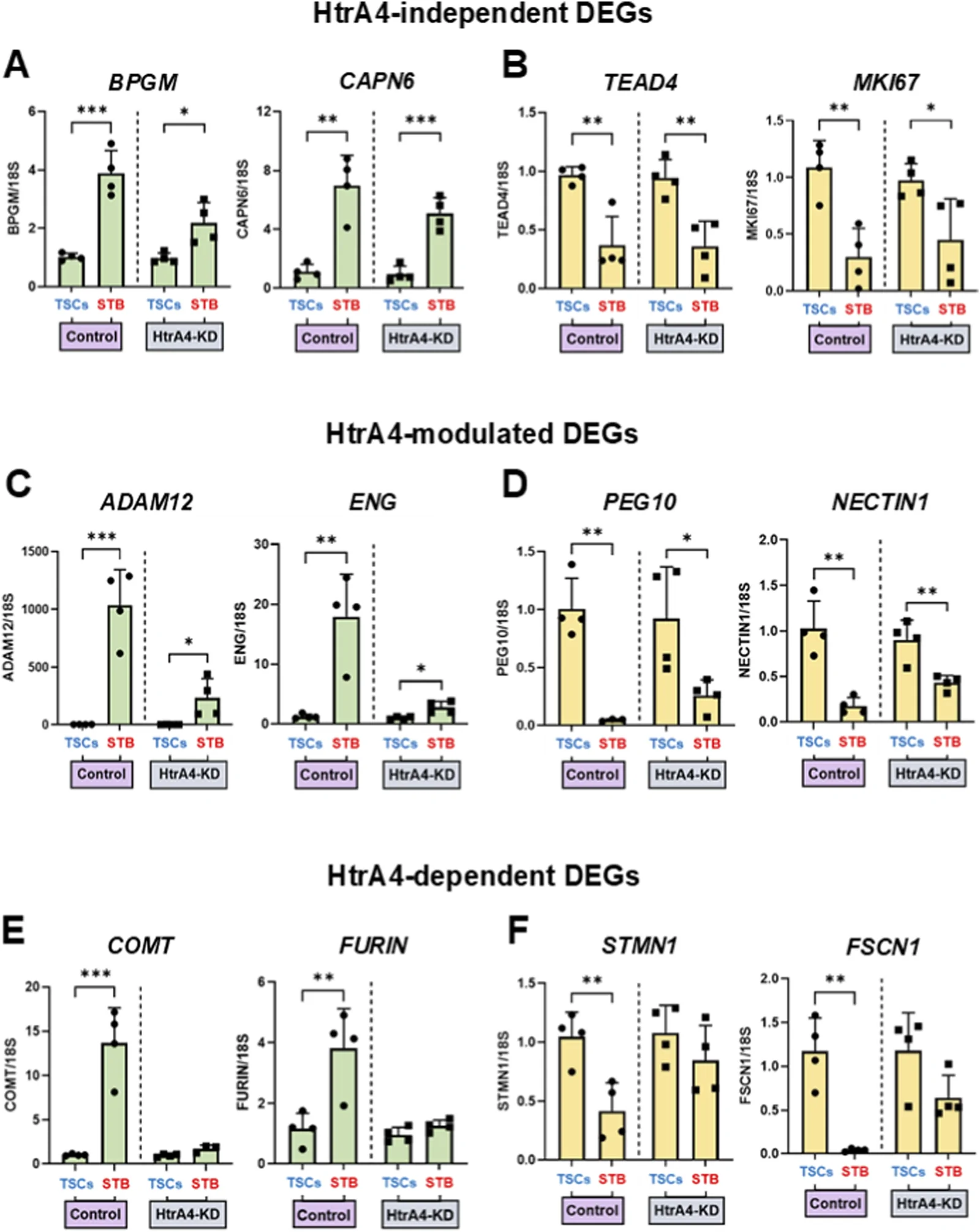
Validation of representative DEGs by RT-PCR analysis in control and HtrA4-KD TSCs upon stimulation for STB differentiation. **(A)** Upregulated and HtrA4-independent (increased similarly in both cell types for STB differentiation): *BPGM* and *CAPN6*. **(B)** Downregulated and HtrA4-independent (reduced similarly in both cell types): *TEAD4* and *MKI67*. **(C)** Upregulated and HtrA4-modulated (increased more profoundly in control than in HtrA4-KD cells): *ADAM12* and *ENG*. **(D)** Downregulated and HtrA4-modulated (reduced more severely in control than in HtrA4-KD cells): *PEG10* and *NECTIN1*. **(E)** Upregulated and HtrA4-dependent (increased in control but not in HtrA4-KD cells): *COMT* and *FURIN*. **(F)** Downregulated and HtrA4-dependent (reduced in control but not in HtrA4-KD cells): *STMN1* and *FSCN1*. Data expressed as mean ± SD; n = 4. *P ≤ 0.05, **P ≤ 0.01, ***P ≤ 0.001.

For upregulated HtrA4-modulated DEGs, mRNA levels of ADAM metallopeptidase domain 12 (*ADAM12)* and vascular endothelium glycoprotein endoglin (*ENG*) were validated (Figure 2C). Both rose significantly in both control and HtrA4-KD cells upon stimulation for STB differentiation, but their increases were greater in control than HtrA4-KD cells (Figure 2C). For downregulated HtrA4-modulated DEGs, paternally expressed 10 (*PEG10*) and cell adhesion molecule Nectin1 (*NECTIN1*) were analyzed (Figure 2D). Both showed significant decreases upon stimulation for STB differentiation in both cell types, though the reduction was greater in control than in HtrA4-KD cells (Figure 2D).

For upregulated and HtrA4-dependent DEGs, catechol-O-methyltransferase (*COMT*) and serine endopeptidase furin (*FURIN*) were tested (Figure 2E), and mRNA levels of both genes increased markedly upon stimulation for STB differentiation in controls but remained unchanged in HtrA4-KD cells. For downregulated HtrA4-dependent DEGs, cytosolic negative regulator of microtubule assembly Stathmin 1 (*STMN1*) and fascin actin-bundling protein (*FSCN1*) were examined (Figure 2F); both were significantly reduced in control cells but not in HtrA4-KD cells. Overall, these RT-PCR results were consistent with the RNA-seq data.

### Validation of representative DEGs by immunofluorescence

We further validated the changes of TEAD4, ENG and FURIN at the protein level by immunofluorescence staining in control and HtrA4-KD TSCs cultured with and without stimulation for STB differentiation (Figure 3); they represented HtrA4-independent, HtrA4-modulated, and HtrA4-dependent DEGs, respectively.

**FIGURE 3:**
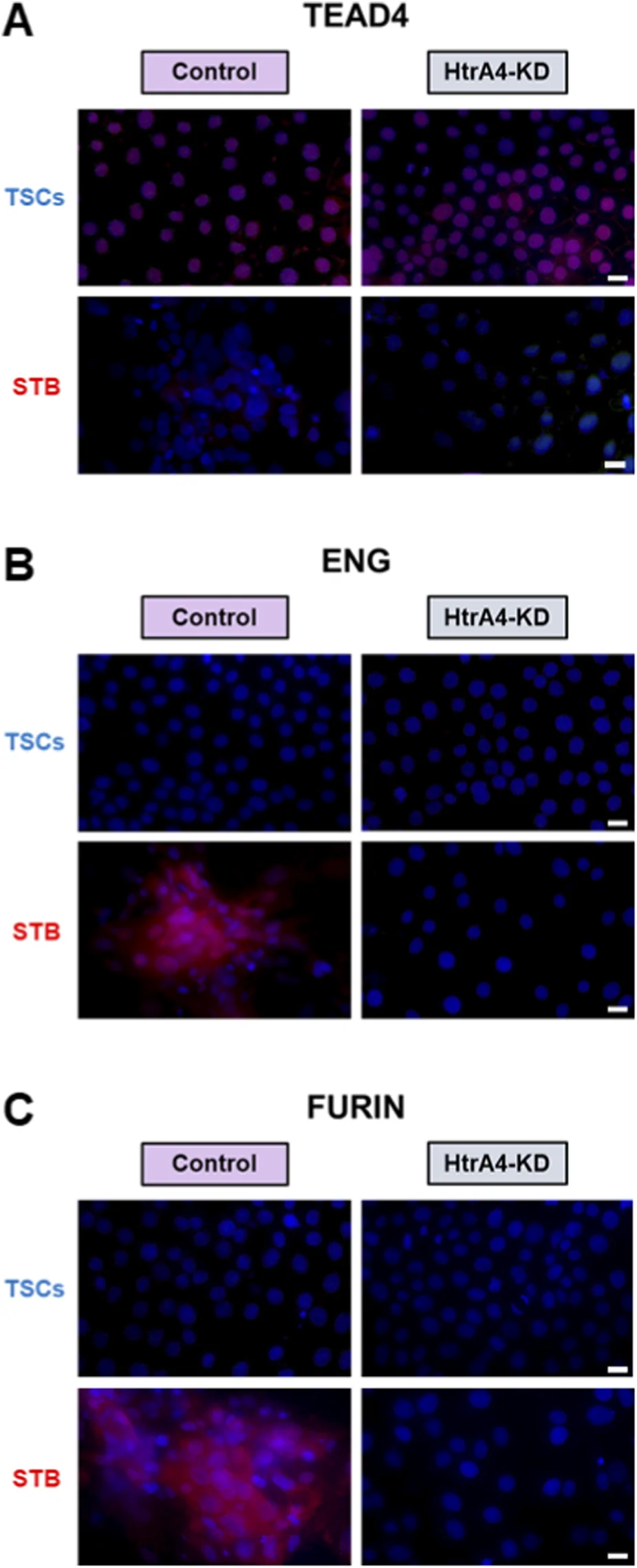
Validation of representative DEGs by immunofluorescence in control and HtrA4-KD TSCs upon stimulation for STB differentiation. **(A)** TEAD4 (red), downregulated and HtrA4-independent for STB differentiation. **(B)** ENG (red), upregulated and HtrA4-modulated. **(C)** FURIN (red), upregulated and HtrA4-dependent. Nuclei counterstained with DAPI (blue), 40x magnification. Representative images are shown, n = 4.

TEAD4 protein was localized to the nuclei of TSCs of both control and HtrA4-KD groups, and levels decreased drastically after stimulation for STB differentiation in both cell types (Figure 3A), consistent with TEAD4 being downregulated and HtrA4-independent. The protein of ENG, a representative of upregulated HtrA4-modulated DEGs, was below detection in TSCs of both cell types, but was clearly positive in cells stimulated for STB differentiation in the control and remained low in HtrA4-KD cells (Figure 3B). FURIN, which was categorized as being upregulated and HtrA4-dependent, was below detection in both TSCs, but intensely positive in control but not in HtrA4-KD cells upon stimulation for STB differentiation (Figure 3C). Overall, these observations at the protein level further validated the RNA-seq and RT-PCR results.

### Functional and pathway analyses of DEG sub-groups in STB differentiation

We next analyzed the genes of the above six sub-categories of DEGs of control cells to identify functions and pathways enriched within each sub-category, thereby understanding the main cellular processes of STB differentiation that were HtrA4-independent, HtrA4-modulated, and HtrA4-dependent. For each DEG category, we searched for enriched GO and KEGG terms using the DAVID tool, then scrutinized the top 15 terms and displayed them graphically, showing their significance and number of genes represented within each term (Figures 4–6). Furthermore, for each sub-category, a summative interaction map was constructed to illustrate the interconnections of the enriched terms (Figures 4–6).

**FIGURE 4:**
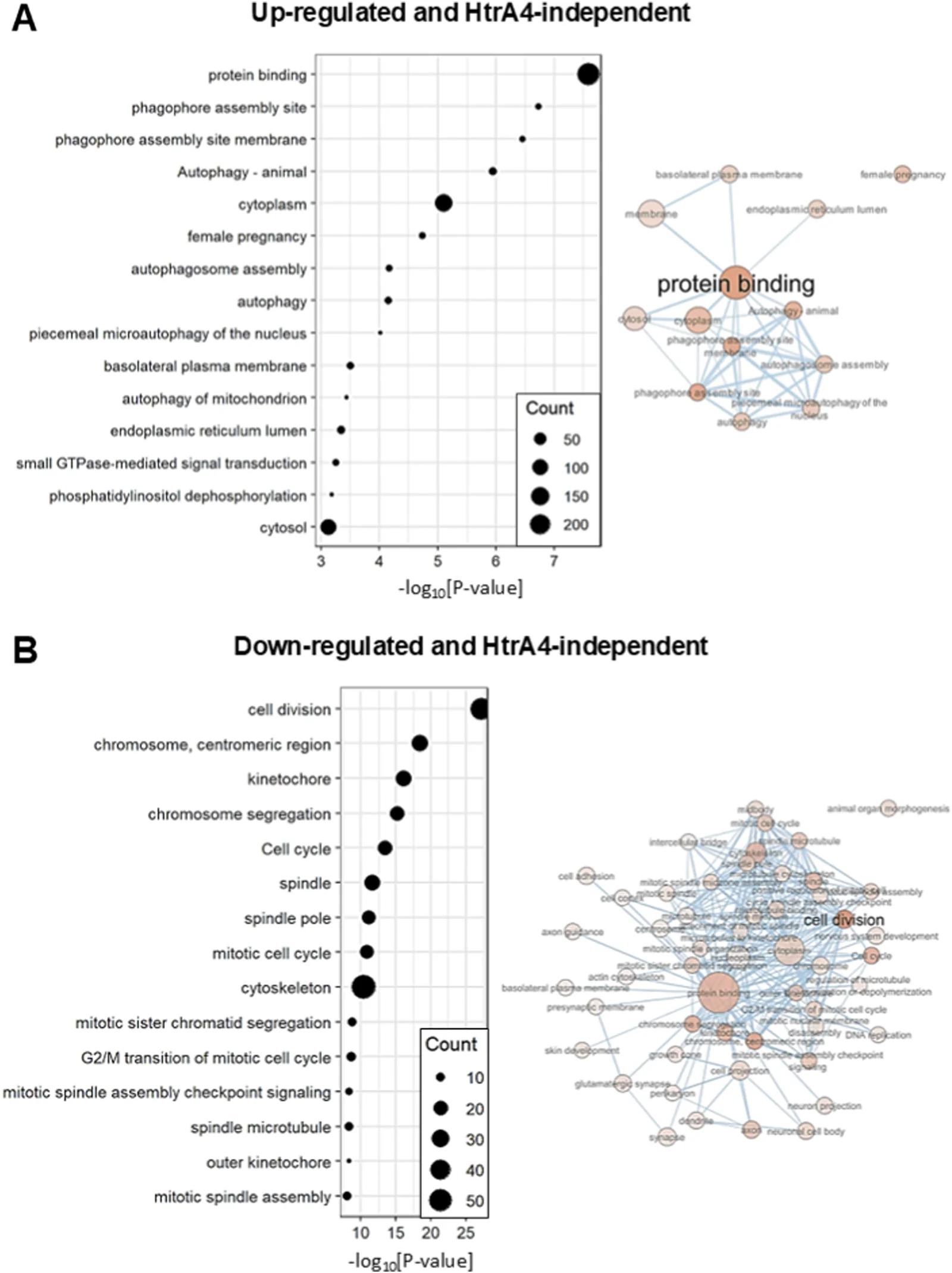
GO and KEGG knowledgebase analysis of HtrA4-independent DEGs during STB differentiation. Enriched GO terms and KEGG pathways showing significant overlap with **(A)** upregulated and HtrA4-independent DEGs and **(B)** downregulated and HtrA4-independent DEGs. For each, the dot plot on the left displays the top 15 ranked GO and KEGG terms, with gene counts indicated by dot sizes and significance by (-log_10_ [P-value], higher values indicate greater significance) on the X-axis. The interaction map on the right illustrates the interactions and overlaps of these enriched terms, where each node represents an enriched term with its size dictated by gene counts and color by significance (with darker shades indicating higher significance), while connecting edges denote shared genes between pathways to highlight core biological modules.

**FIGURE 5:**
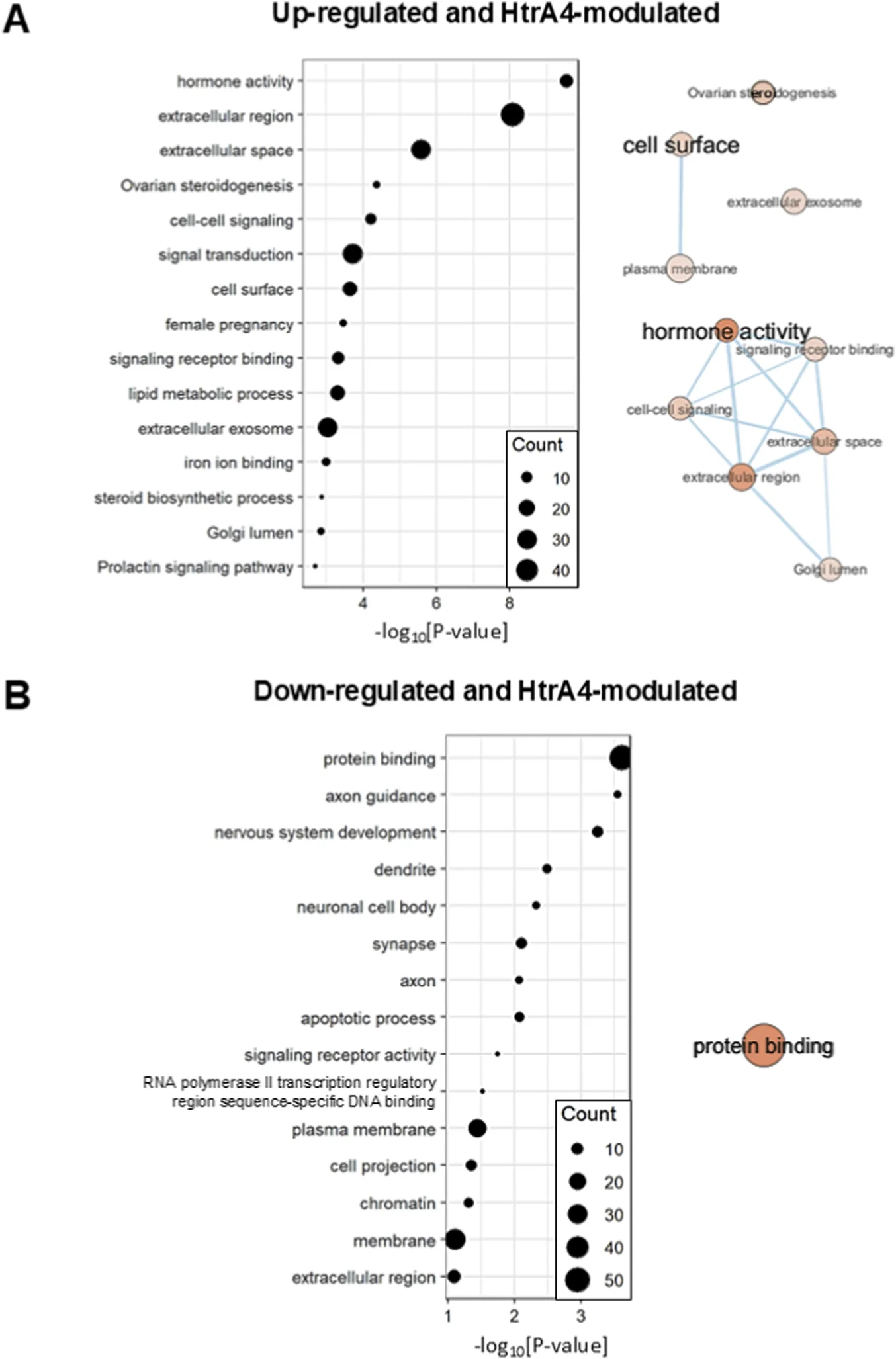
Pathway analysis of HtrA4-modulated DEGs for STB differentiation. Enriched GO terms and KEGG pathways with significant overlap with **(A)** upregulated and HtrA4-modulated DEGs and **(B)** downregulated and HtrA4-modulated DEGs. For each, the dot plot on the left displays the top 15 ranked GO and KEGG terms, and the interaction map on the right illustrates the interactions and overlaps of these enriched terms. Other details are as described in Figure 4.

**FIGURE 6:**
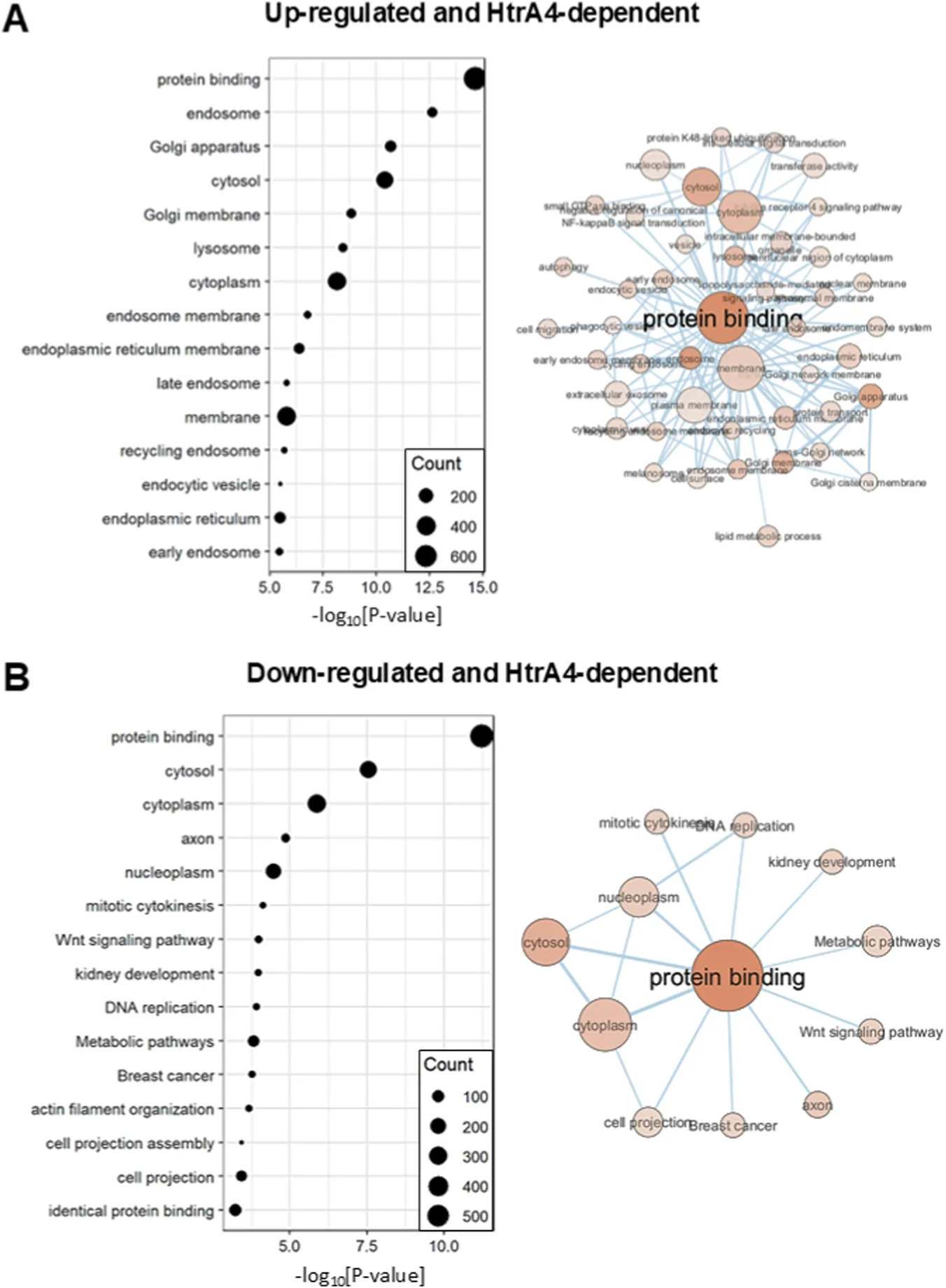
Pathway analysis of HtrA4-dependent DEGs for STB differentiation. Enriched GO terms and KEGG pathways with significant overlap with **(A)** upregulated and HtrA4-dependent DEGs and **(B)** downregulated and HtrA4-dependent DEGs. For each, the dot plot on the left displays the top 15 ranked GO and KEGG terms, and the interaction map on the right illustrates the interactions and overlaps of these enriched terms. Other details are as described in Figure 4.

#### HtrA4-independent DEGs are primarily associated with early syncytialization events

Figure 4A shows the analysis of upregulated HtrA4-independent DEGs (296 genes in total). The three most significantly enriched terms included protein binding, phagophore assembly site, and phagophore assembly site membrane (Figure 4A), alongside several terms related to autophagy, cellular compartments, female pregnancy, and signaling (full list shown in Figure 4A). Among these, protein binding, cytoplasm, and cytosol were the most heavily enriched, with each containing more than 100 genes (Figure 4A). The interaction map, shown on the right-hand side, indicated extensive interconnections centered on protein binding, with major nodes corresponding to autophagy, autophagosome assembly, piecemeal microautophagy of the nucleus, and cellular membranes (Figure 4A). The prominent representation of autophagy-related terms is consistent with the increase of autophagy as a crucial part of syncytialization (Singh et al., 2024). Our data thus suggest that the initial steps of autophagy were HtrA4-independent, as these pathways occurred similarly in both control and HtrA4-KD cells.

On the other hand, the downregulated HtrA4-independent DEGs (290 genes in total) were enriched with entirely different pathways (Figure 4B). The top three terms identified included cell division, chromosome/centromeric region, and kinetochore (Figure 4B). Other terms included chromosome segregation, cell cycle, spindle/spindle pole, mitotic cell cycle, alongside cytoskeleton (which contained the most genes), mitotic sister chromatid segregation, G2M transition of mitotic cell cycle, mitotic spindle assembly/checkpoint signaling, spindle microtubule, and outer kinetochore (Figure 4B). The interactive map displayed massive interconnections among these pathways around cell division (Figure 4B). These results are in line with the current understanding that syncytialization requires cells to halt proliferation and exit from cell cycle to acquire a fusion-competent state (Li et al., 2023), and our findings suggest that such cell cycle processes were HtrA4-independent, as they occurred in both control and HtrA4-KD cells upon stimulation for STB differentiation.

#### HtrA4-modulated DEGs largely involve hormone biosynthesis and plasma membrane remodeling

Figure 5A shows the GO and KEGG terms significantly enriched in upregulated HtrA4-modulated DEGs (163 genes in total). The three most significant terms were hormone activity, extracellular region and extracellular space; other terms included ovarian steroidogenesis, cell-cell signaling, cell surface, signaling receptor binding, lipid metabolic process, alongside extracellular exosome, iron ion binding, Golgi lumen and prolactin signaling pathway (Figure 5A). Terms of extracellular region/space, exosome and signal transduction were enriched with relatively more genes. The interaction map highlighted hormone activity, cell surface/plasma membrane, extracellular region/space, cell-cell signaling, and signaling receptor binding as major interactive hubs (Figure 5A). These results are highly consistent with the knowledge that syncytialization induces hormone biosynthesis and secretion, dramatic plasma membrane remodeling, significant alterations of extracellular matrix (ECM)/signaling, and major changes in communications with adjacent cells (Li et al., 2023; Zhou et al., 2023). Our analysis implies that these important cellular processes of STB differentiation are HtrA4-modulated since they were attenuated in HtrA4-KD cells.

For downregulated and HtrA4-modulated DEGs (63 genes in total), their major enriched GO and KEGG terms are shown in Figure 5B. The top pathway was protein binding, which was followed by a number of pathways related to the nervous system including axon guidance, nervous system development, dendrite, neuronal cell body, synapse, axon, cell projection; other terms included apoptotic process, signal receptor activity, plasma membrane, membrane, and extracellular region (Figure 5B). Among these, protein binding, plasma membrane, and membrane were enriched with relatively more genes. The search for interconnections found protein binding as the only major node (Figure 5B), highlighting that protein binding is the predominant theme at this juncture, although this could also be due to the relatively smaller number of genes present in this sub-category. Nonetheless, protein binding is critically involved in nearly all cellular activities, and its importance in syncytialization is well documented (Zhou et al., 2023; Li et al., 2025). Our results suggest that HtrA4 is partially involved in reducing protein binding, which is not surprising as proteases can activate or inactivate protein interactions through protein cleavage (Bleuez et al., 2022). Furthermore, our data indicate that biological processes and pathways related to nervous system are suppressed during STB differentiation, and such suppression is HtrA4-modulated.

#### HtrA4-dependent DEGs relate predominantly to endomembrane processes and structural remodeling required for cell fusion

For upregulated HtrA4-dependent DEGs (819 genes), their enriched GO and KEGG terms are shown in Figure 6A. The top three terms were protein binding, endosome and Golgi apparatus. Other significantly overrepresented terms included cytosol, Golgi membrane, lysosome, cytoplasm, endosome membrane, ER membrane, recycling endosome, endocytic vesicle and early endosome (Figure 6A). The interaction map illustrated protein binding again as the interconnection hub, which was interwoven with sub-hubs such as cytoplasm, cytosol and membrane (Figure 6A), consistent with all three terms being enriched with relatively more genes than other terms. A particular observation here was the overrepresentation of terms related to endosome (such as endosome membrane, early/late/recycling endosome, and endocytic vesicle) and associated components (such as the Golgi apparatus, ER, and lysosome). These results are consistent with the literature that endosomal activities are expanded during syncytialization for nutrient transport and intercellular communication (Fan et al., 2024). Our analysis suggests that such essential STB differentiation processes were HtrA4-dependent because these were inhibited in HtrA4-KD cells.

Figure 6B shows the pathways enriched by DEGs that were downregulated and HtrA4-dependent (694 genes) (Figure 6B). The top three terms were protein binding, cytosol and cytoplasm (Figure 6B). More specific terms included axon, mitotic cytokinesis, Wnt signaling pathway, metabolic pathways, actin filament organization, cell projection/assembly, and identical protein binding, together with more generic terms such as nucleoplasm, kidney development and breast cancer (Figure 6B). The interaction map illustrates protein binding once again as the interconnection hub, which radiates to smaller intersecting nodes such as cytosol, cytoplasm and nucleoplasm (Figure 6B). Decreasing activities in these regions/pathways are again known to be part of STB differentiation in remodeling various components of the cytoplasm and nucleoplasm to acquire a multinucleated cell morphology. Moreover, reductions in certain metabolic pathways are needed for terminal STB differentiation to divert cellular energy to endocrine and barrier functions (Kol et al., 2017; Bucher et al., 2021). Additionally, a noteworthy GO term enriched here was reduction of identical protein binding (Figure 6B), such as the dimerization of cadherin molecules (Chen et al., 2005), which is a critical mechanism by which cell adhesion and junctional proteins are modulated to allow inter-cellular mixing and cell fusion during STB differentiation (Zhou et al., 2023). Our analysis indicated that these crucial STB differentiation processes were significantly HtrA4-dependent because they were blocked in HtrA4-KD cells.

#### DEGs unique to HtrA4-KD cells show distinct functional pathways from controls

Molecular pathways associated with DEGs that arose uniquely in HtrA4-KD cells upon stimulation for STB differentiation were also analyzed (Supplementary Figure S1). The few DEGs that changed to a greater extent in HtrA4-KD than in control cells were included here, as the number was too low for independent analysis. As before, upregulated (259 plus 11 as one group) and downregulated (275 plus 16) candidates were examined separately.

For the upregulated DEGs, ECM was the top enriched pathway (Supplementary Figure S1A); intriguingly, other terms were dominated by cellular responses to minerals such as copper ion, cadmium ion and zinc ion, concerning their absorption, detoxification and homeostasis. Also on the list were negative regulation of growth, focal adhesion, ECM-receptor interaction, human papillomavirus infection, basement membrane, protein binding, relaxin signaling, and FoxO signaling (Supplementary Figure S1A). Notably, protein binding was enriched with the largest number of genes (Supplementary Figure S1A).

For the downregulated DEGs, protein binding stood out as the top term, which also contained the greatest number of genes (Supplementary Figure S1B). Other major terms included single-stranded DNA helicase activity, cytosol, negative regulation of BMP signaling pathway, ERK1 and ERK2 cascade, establishment of localization in cell, and protein kinase binding. Additional pathways identified related to cilium assembly, centrosome, protein homodimerization activity, receptor ligand activity, intercellular bridge, epithelial to mesenchymal transition, signal transduction, and positive regulation of apoptotic process (Supplementary Figure S1D).

Overall, apart from protein binding and cytosol, these pathways are largely discrete from those identified in control cells, consistent with the notion that unique DEGs of HtrA4-KD cells were likely reflective of cellular counterbalancing responses to potentially overcome HtrA4 deficiency and the differentiation block.

### SEM confirmation of impairment of cell fusion and multinucleation in HtrA4-KD cells

To further validate that HtrA4-KD cells were responsive to the stimulation of STB differentiation but unable to fully accomplish the process, we next employed SEM and closely examined the morphological changes of control and HtrA4-KD TSCs upon stimulation for STB differentiation, thereby identifying when and how HtrA4-KD cells were impeded during this fusion process. Control and HtrA4-KD cells were cultured to form a monolayer, then stimulated for STB differentiation for 6 days as before, but cellular topography and overall morphology were examined on days 0, 2, 3, 4, and 6 after the stimulation (Figure 7A).

**FIGURE 7:**
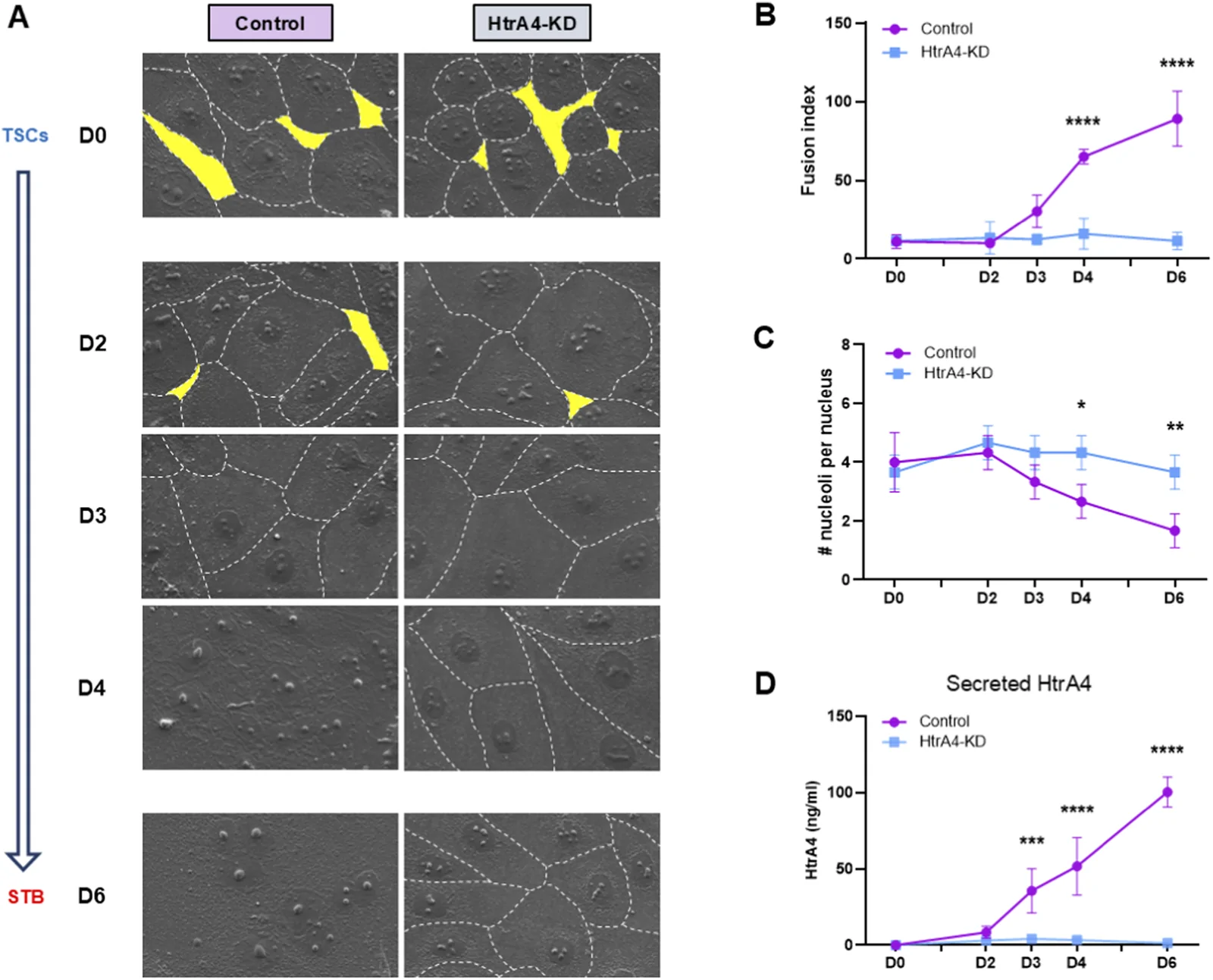
Scanning electron microscopy (SEM) analysis of control and HtrA4-KD TSCs along the course of stimulation for STB differentiation. **(A)** Cell-cell interactions and surface morphology of control and HtrA4-KD cells on day **(D)** 0, 2, 3, 4 and 6 after stimulation for STB differentiation. Cell membranes are indicated by white dashed lines, and gaps between cells highlighted in yellow. Cell nuclei: dark and round regions in the cytoplasm; nucleoli: protrusions within nuclei. Images taken at 2000x magnification. **(B)** Fusion index, **(C)** Average number of nucleoli per nucleus, and **(D)** Secreted levels of HtrA4 in culture media along the time course following stimulation for STB differentiation. Comparisons in all were between Control and HtrA4-KD at each timepoint (n = 3). *P ≤ 0.05, **P ≤ 0.01, ***P ≤ 0.001, ****P ≤ 0.0001.

Initially, on days 0 and two following the stimulation, control and HtrA4-KD cells appeared similar in gross morphology; both cell types were mononuclear and cuboidal with round cell boundaries (Figure 7A). Gaps between cells were noticeably reduced by day 2 (highlighted in yellow in Figure 7A), which was apparent for both cell types (Figure 7A). By day 3, intercellular gaps were rarely seen in either cell type, while cell boundaries began to appear more polygonal (Figure 7A). From here onwards, control and HtrA4-KD cells started to deviate from each other (Figure 7A). The control cells began to lose cell boundaries and commenced fusion, and by day 6 the large majority of them became multinucleated (Figure 7A). In contrast, HtrA4-KD cells showed no further changes, with individual cells remaining polygonal in shape and still being surrounded by clear borders even by day 6 (Figure 7A).

We also inspected the number of nuclei present in individual cells, and quantified cell fusion index by calculating the percentage of nuclei present within multinucleated cells relative to total nuclei (Figure 7B). In the first 2 days, no significant fusion was apparent in either control or HtrA4-KD cells. From day 3 onwards, control cells started to increase in fusion index and continued to rise following this point (Figure 7B); in contrast, HtrA4-KD cells showed no change in fusion index throughout (Figure 7B). As such, the difference between the two cell types became increasingly more significant over time (Figure 7B).

We also noticed clear changes in the number of nucleoli present in individual nuclei, which were also quantified (Figure 7C). During the first 2 days upon stimulation for STB differentiation, the number of nucleoli per nucleus increased slightly but not significantly in both cell types; from day 3 onwards, while the control began to decrease and continued to decline, HtrA4-KD cells remained constant until day 4, then declined only slightly by day 6 (Figure 7C). Therefore, the 2 cell types differed significantly from day 4 onwards in nucleolus number (Figure 7C). The timing of these changes/differences coincided with the changes in fusion index shown in Figure 7B.

We also assessed how HtrA4 production and secretion changed over time in control and HtrA4-KD cells (Figure 7D). On days 0 and 2, HtrA4 was very low in both cell types with no significant differences (Figure 7D). However, the control started to increase HtrA4 secretion after day 2 and continued the climb profoundly thereafter (Figure 7D). In contrast, HtrA4-KD cells displayed no significant elevations in HtrA4 throughout (Figure 7D). Thus, HtrA4 levels differed between the 2 cell types more significantly over time. The timing of HtrA4 surge in the control, and differences between the 2 cell types, corresponded well with the changes/differences in cell morphology, fusion index, and nucleoli shown in Figures 7A–C. It is also worth noting that the HtrA4 dynamics revealed here over the time course of STB differentiation, expanded our previous observations that HtrA4 is significantly elevated when TSCs are differentiated for 6 days into STB and that such elevations are blocked in HtrA4-KD cells (Thach et al., 2024).

## Discussion

HtrA4 is exclusively produced by placentas of humans and higher primates, and its over-production is strongly associated with preeclampsia, yet its fundamental functions in human placentation remain elusive (Wang and Nie, 2021). Building on our previous work demonstrating that HtrA4 is obligatory for trophoblast differentiation, the current study was undertaken to understand how HtrA4 is involved in STB differentiation. We compared the global transcriptomic changes displayed by control and HtrA4-KD TSCs upon stimulation for STB differentiation, to identify the deficiencies of HtrA4-KD cells in achieving gene expression profiles characteristic of genuine STB differentiation observed in control cells. While both types of TSCs altered their protein-coding genes considerably towards STB differentiation, the changes were much less pronounced in HtrA4-KD cells where HtrA4 was suppressed. Further analysis revealed that among the significant DEGs associated with authentic STB differentiation in controls, 25% of these also occurred in HtrA4-KD cells, indicating these DEGs were HtrA4-independent; in contrast, the remaining 75% DEGs were either severely attenuated or completely blocked in HtrA4-KD cells, signifying that they were HtrA4-modulated or HtrA4-dependent. Pathway analysis of these distinct DEGs suggested that early STB differentiation events, such as halting cell division (e.g., ceasing chromosome segregation and G2M transition) and initiation of autophagy (e.g., assembly of phagophore and autophagosome), were independent of HtrA4. However, many subsequent processes of STB differentiation were either modulated by or dependent on HtrA4 action, some of which included hormone activity, remodeling of cell surface membrane, alterations in extracellular region/matrix, adjustments in cell-cell signaling, expansion of endosome activities, restructuring of various components of the cytoplasm and cytoskeleton, and tempering of metabolic pathways. Intriguingly, our data imply that pathways related to nervous system development were inhibited during STB differentiation, and this repression was influenced by HtrA4. Furthermore, altering protein binding was repeatedly identified as a major pathway of HtrA4 influence. Finally, through SEM, we examined morphological changes of TSCs during the time course of STB differentiation and visually demonstrated that while HtrA4-KD cells initiated the differentiation process, they failed to progress to terminal cell fusion into the multinucleated STB. Collectively, these studies provide important new insights into the pivotal role of HtrA4 in STB differentiation.

Our study first established the DEGs displayed in the control cells as characteristic gene expression changes of STB differentiation, then compared them to HtrA4-KD cells to identify differentiation defects and deduce how the process was influenced by HtrA4. This led to the grouping of control DEGs associated with normal STB differentiation into six sub-categories. These classifications are based on comparative transcriptomic changes and do not necessarily imply that these genes are direct targets of HtrA4, as the effects could be direct or indirect. Subsequent analysis of the processes and pathways enriched by these DEG sub-groups indicated that they were indeed largely consistent with the known characteristics of various syncytialization stages, validating our strategy of DEG grouping for genuine STB differentiation, and providing insights into the positioning of HtrA4 action. Subsequently, we analyzed the top pathways signified by each sub-group to understand how STB differentiation was influenced by HtrA4.

Among processes identified as upregulated and HtrA4-independent, initiation of autophagy predominated. This conserved cellular degradation process facilitates the orderly recycling of cellular materials following cell fusion, starting with the assembly of specialized membranes termed phagophore, which then engulf cytoplasmic content to produce autophagosome vesicles for eventual lysosomal degradation (Singh et al., 2024). Several studies have reported that STB exhibits elevated levels of autophagy activities (Singh et al., 2024), and a recent report using electron microscopy further revealed that high autophagic flux may be a visual indicator of early syncytialization (Tozawa et al., 2025). Our results are thus not only consistent with prior knowledge, but also identified that these early events of STB differentiation were independent of HtrA4.

For this sub-category of DEGs, *BPGM* and *CAPN6* were validated as representatives by RT-PCR. The multifunctional enzyme BPGM is reported to be restricted to human erythrocytes and STB (Stroganov et al., 2024). BPGM is localized to the apical side of the STB within the placenta, and is posited to play a potential role in aiding oxygen transfer to fetal systems (Stroganov et al., 2024). Meanwhile, CAPN6 is known to be involved in several cellular processes including microtubule stabilization, cell movement and inhibition of apoptosis (Dear and Boehm, 1999; Chen et al., 2020), all of which are required for STB differentiation.

For pathways categorized as downregulated and HtrA4-independent, the leading GO terms were consistently related to cell division and mitotic cell cycle. This is in accordance with our understanding that for fusion to proceed, cells must exit from the mitotic cell cycle and suppress factors associated with stemness and proliferation, such as *TEAD4* (Saha et al., 2020) and *MKI67* (Sun and Kaufman, 2018). In the placenta, TEAD4 is important in promoting trophoblast self-renewal and placental maintenance during pregnancy (Saha et al., 2020). MKI67 is a marker of active cell division and proliferation (Sun and Kaufman, 2018); fittingly, MKI67 is detected in CTB but not in STB (Lee et al., 2018). The mRNA levels of both *TEAD4* and *MKI67* were confirmed to be significantly reduced in both control and HtrA4-KD TSCs upon stimulation for STB differentiation, and reductions in TEAD4 protein were further verified. Together, these results validated the RNAseq data, and supported the notion that HtrA4 was dispensable for cells to instigate commitment to syncytialization.

For pathways categorized as upregulated and HtrA4-modulated, hormone biosynthesis and secretion were among the top components. This is again in line with the established understanding that the STB produces numerous placental hormones and growth factors to modulate the maternal physiology (Liu et al., 2018). *ADAM12* and *ENG* (endoglin) were validated by RT-PCR as two representatives of this sub-group of DEGs, and both were significantly upregulated in both types of TSCs upon stimulation for STB differentiation, though the magnitude was considerably attenuated in HtrA4-KD cells. The disintegrin ADAM12, detected in both CTB and STB in the placenta with a higher level in the STB, is proposed to direct remodeling of intercellular boundaries to potentiate trophoblast fusion (Aghababaei et al., 2015). ENG, also a product of STB, regulates placental differentiation and invasion during pregnancy (Wang et al., 2016), and its soluble form has etiological relevance in the pathogenesis of preeclampsia (Tariq et al., 2025). Moreover, we have previously demonstrated that STB differentiation of control TSCs is accompanied by considerable production of pregnancy hormone hCG, but this is severely attenuated in HtrA4-KD cells (Thach et al., 2024). Reflecting this, the hCG β-subunit genes (*CGB1*-*8*) were identified in this sub-group. Together, these results imply that HtrA4 may act as a necessary amplifier for endocrine maturation during STB differentiation.

Pathways identified as downregulated and HtrA4-modulated were dominated by protein binding and were unexpectedly overrepresented by nervous system-related processes, which included GO terms such as axon guidance, dendrite, neuronal cell body, and synapse. This was evidenced by the downregulation of several master regulators of neuronal differentiation such as *LHX2* (Hou et al., 2013). It could be envisaged that downregulation of such genes associated with nervous system development may ensure lineage specification of TSC differentiation towards STB. As HtrA4 was identified to partially influence these pathways, it is tempting to speculate that HtrA4 may be required to safeguard the specificity of STB differentiation. *PEG10* and *NECTIN1* were validated by RT-PCR as two representative DEGs of this sub-group; both were significantly downregulated in both types of TSCs upon stimulation for STB differentiation, though the reduction was milder in HtrA4-KD than control cells. PEG10 is involved in early placentation and trophoblast cell lineage specification (Abed et al., 2019). NECTIN1, a cell-adhesion molecule, is commonly found in epithelial cells and known to regulate adherens and tight junctions, cell polarization, and migration (Takai et al., 2008).

Intriguingly, although the up- and downregulated and HtrA4-modulated DEGs were analyzed independently, both categories were strongly enriched with pathways involving molecular interactions at the plasma membrane and extracellular region. Related GO terms included extracellular region/space, cell-cell signaling, cell surface, signal receptor binding, protein binding, and signaling receptor activity. These findings therefore indicate that HtrA4 is a necessary regulator of plasma membrane and extracellular functions during STB differentiation.

For pathways identified as upregulated and HtrA4-dependent, the endomembrane system and endocytic pathways stood out. The identification of such systems for normal STB differentiation is in line with the functional importance of STB in placental nutrient transfer and waste removal (Li et al., 2025). This is also in accordance with the knowledge that STB differentiation and hormonal release require the expansion and optimal function of the endomembrane system, such as the Golgi apparatus, ER, and their organelle machinery (Fan et al., 2024). In addition to these known elements of syncytialization, our analysis now suggests that HtrA4 is indispensable for these important processes during STB differentiation. *FURIN* and *COMT* were validated by RT-PCR as the two representative DEGs of this sub-group, and FURIN protein was further corroborated by immunofluorescence. FURIN is reported to critically mediate trophoblast syncytialization (Zhou et al., 2013), whereas COMT is involved in hormone metabolism and its levels are decreased in preeclamptic pregnancies (Kanasaki et al., 2008).

Pathways classified as downregulated and HtrA4-dependent were enriched for processes involving the cytosol and cytoplasm, such as mitotic cytokinesis, Wnt signaling, and metabolic pathways. As discussed previously, STB differentiation requires cells to cease proliferation. It is thus fitting that mitotic cytokinesis, the final stage of cell division that divides the cytoplasm, is also downregulated for STB differentiation. Our analysis thus indicates that HtrA4 may influence the repression of late-stage cell division. Wnt signaling, a crucial component of cell stemness, is required for expansion of trophoblast progenitors and maintenance of TSCs in an undifferentiated state, and it must be inhibited for syncytialization (Jaremek et al., 2021). However, this suppression was impaired in HtrA4-KD cells. Together, these data are consistent with HtrA4-KD cells remaining unfused, despite having ostensibly exited from the cell cycle upon stimulation for STB differentiation.

Two representative DEGs of this sub-category, *STMN1* and *FSCN1*, were validated by RT-PCR, and both were downregulated significantly in controls but not in HtrA4-KD cells upon stimulation for STB differentiation. STMN1, a microtubule regulatory protein, is known to be highly expressed in villous CTBs but absent in the STB in first-trimester human placenta (Tian et al., 2015). FSCN1, an actin filament-bundling protein, supports various cell structures beneath the plasma membrane and plays an important role in maintaining cell-matrix adhesion (Li et al., 2022); such activities would need to be reduced during STB differentiation. These data thus suggest that the restructuring of microtubules and actin filaments hinges on the actions of HtrA4 for STB differentiation to progress.

The above analyses revealed that the large majority of cellular processes required for full STB differentiation were HtrA4-dependent. However, some initial events of STB differentiation did occur independent of HtrA4. To complement the above understanding, we also analyzed pathways enriched uniquely by HtrA4-KD cells and identified several interesting terms.

For DEGs upregulated specifically in HtrA4-KD cells, the most prominent terms related to the absorption, detoxification and homeostasis of several divalent cations such as copper and zinc ions. In the context of STB differentiation, these trace minerals are the catalytic cores of key antioxidant enzymes to neutralize free radicals to prevent protein damage and oxidative stress (Mondola et al., 2016). Furthermore, HtrA4-KD cells also upregulated relaxin and FoxO signaling; the former is known to act as a powerful protector against oxidative stress (Waza et al., 2018), and the latter functions as an important sensor of oxidative stress to upregulate antioxidant enzymes (Zhang et al., 2011). Given that HtrA enzymes in bacteria critically protect cells from oxidative stress by removing damaged proteins (Latimer et al., 2026), it could be speculated that HtrA4 may also play a similar role during normal STB differentiation, and that other arms of the cellular antioxidant defense network were intensified to compensate for HtrA4 deficiency. Future experiments are warranted to investigate whether HtrA4 acts as an important antioxidant factor to modulate reactive oxygen species during STB differentiation.

Among the downregulated pathways specific to HtrA4-KD cells were single-stranded DNA helicase activity and centrosomes, likely serving to enforce permanent cell-cycle exit despite differentiation arrest. Other top terms included negative regulation of BMP signaling and the ERK1 and ERK2 cascades. While suppressing these pathways is normally essential for STB differentiation (Roberts et al., 2022; Song et al., 2021), this alteration in differentiation-stalled HtrA4-KD cells may represent a compensatory rescue response to prevent cellular stress and apoptosis.

Morphological analyses of control and HtrA4-KD cells using SEM provided further insights into the involvement of HtrA4 in STB differentiation. Within the first 3 days upon stimulation, both control and HtrA4-KD cells transitioned similarly from a rounder to more polygonal shape, resulting in cells contacting each other very closely at all boundaries with no gaps. This signified that early processes of differentiation occurred to maximize membrane contact and interactions, and that such activities were independent of HtrA4. However, beyond this point, clear differences emerged between the 2 cell types. Structurally, control TSCs progressed with the differentiation to eventually lose cell boundaries and become multinucleated. In contrast, HtrA4-KD cells stalled in their differentiation and remained single-celled and polygonal; which was clearly supported by the fusion index quantification. Furthermore, control cells progressively reduced the number of nucleoli present in each nucleus, a change that was absent in HtrA4-KD cells. These morphological shifts were closely accompanied by continued increases in HtrA4 production and secretion in the control cells, a feature lacking in HtrA4-KD cells. Together, these results provide valuable visual evidence for normal STB differentiation processes and highlight the critical role of HtrA4 in completing the full differentiation process, further corroborating the findings derived from the transcriptomic analysis.

While the above results provide significant insights into the involvement of HtrA4 in STB differentiation, several limitations must be acknowledged. Firstly, 2D cultures of TSCs allowed high-throughput transcriptomic profiling, but they lack the complex paracrine signals of other cell types that would normally be present in the placenta. Secondly, while our transcriptomic analysis focused on global gene changes, it did not account for heterogeneity inherent to a multinucleated syncytium. Thirdly, previous studies using organoids derived from TSCs indicated the existence of an intermediate STB precursor state, which was believed to be nearly absent *in vivo* (Shannon et al., 2024); the approach employed here cannot distinguish between these precursor stages and terminally differentiated STB. However, the samples showed high fusion indexes, suggesting extensive multinucleation occurred and only a low proportion of cells might be in this precursor state if they existed. Fourthly, our current study primarily uncovered correlations in gene expression changes and morphological phenotypes. While these results are invaluable in identifying the broad involvement of HtrA4 in syncytialization, the study was not designed for pinpointing specific substrates and targets of HtrA4 action; future investigations using other techniques such as proteomics or phosphoproteomics are required to elucidate these. Moreover, because its expression is restricted to the placenta, HtrA4 represents a promising candidate for placenta-targeted drug delivery and therapies for pregnancy complications where HtrA4 is significantly increased (Chen and Dong, 2025).

In summary, this study has demonstrated that HtrA4 is dispensable for early events of STB differentiation but is required for many other cellular processes for cells to fully develop into the STB. Modulation of protein binding was identified as a major functional feature of HtrA4, and data also imply that HtrA4 may participate in counteracting oxidative stress during STB differentiation. These findings considerably extend our understanding of STB formation and the pivotal role of HtrA4 in enabling this essential trophoblast differentiation.

## Data Availability

The datasets presented in this study can be found in online repositories. The names of the repository/repositories and accession number(s) can be found below: https://www.ncbi.nlm.nih.gov/geo/, GSE334021.
