# Supplementary material for: Understanding the involvement of HtrA4 during syncytiotrophoblast differentiation
Source: Front Cell Dev Biol. 2026 Sep 29;14:1926149. doi: 10.3389/fcell.2026.1926149 (PMC13658180; doi:10.3389/fcell.2026.1926149)
Supplement: Supplementary file 1 [file Image1.pdf]

A

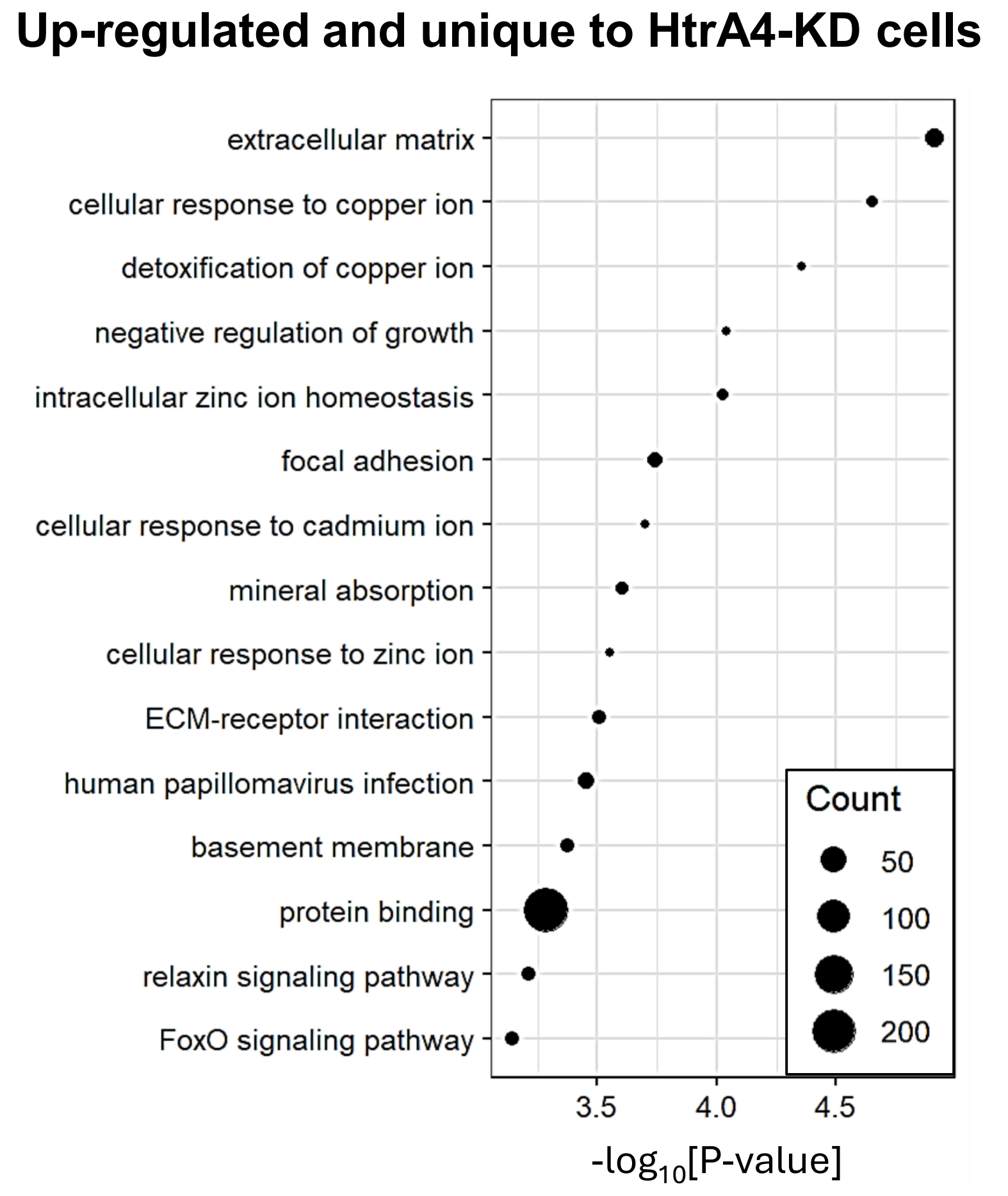

B

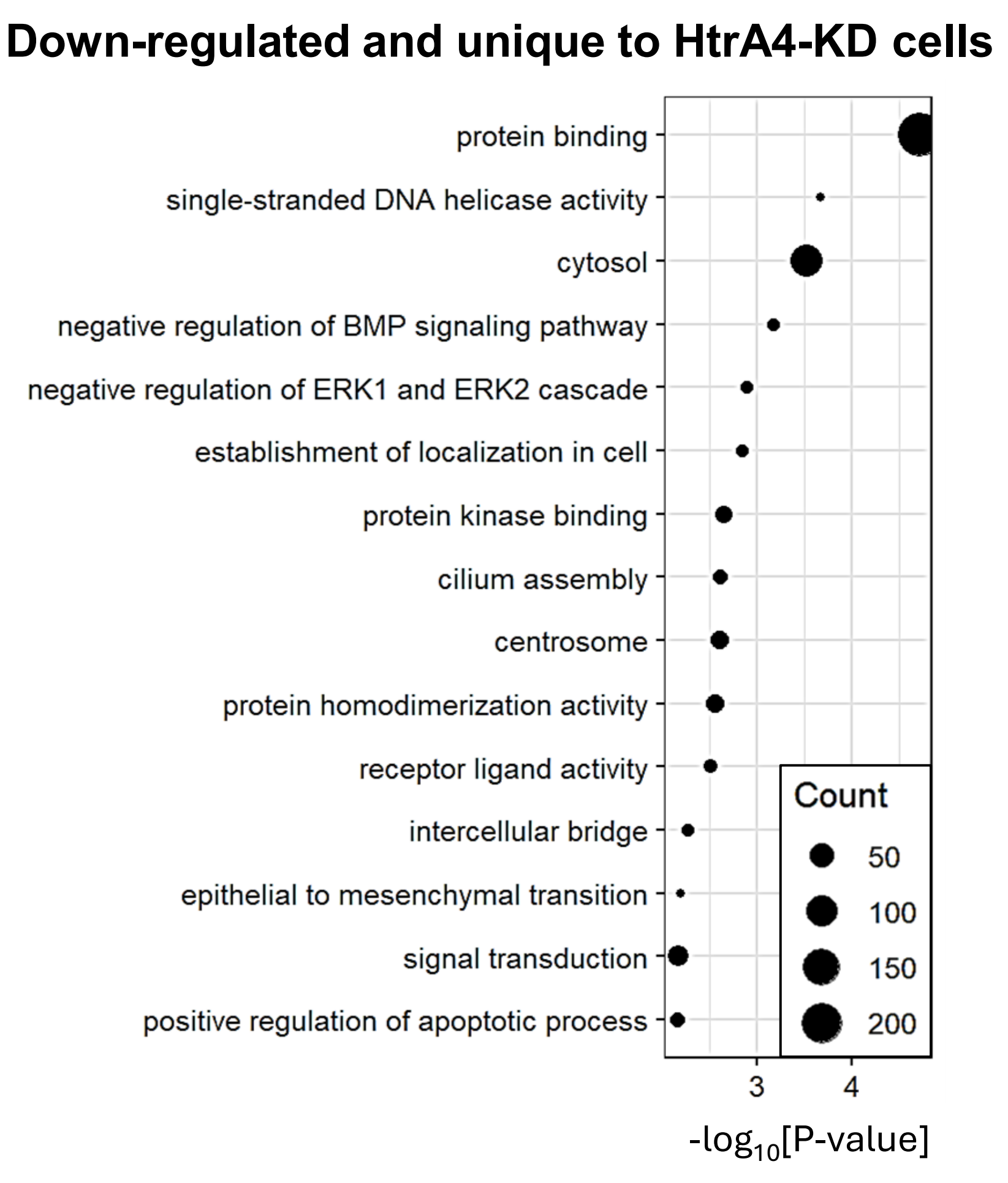

Supplementary Figure S1

Supplementary Figure 1. GO and KEGG knowledgebase analysis of DEGs unique to HtrA4-KD cells during stimulation for STB differentiation. Enriched GO terms and KEGG pathways showing significant overlap with DEGs which were **A)** up-regulated and **B)** down-regulated in HtrA4-KD cells to a greater extent in HtrA4-KD cells than in controls, or only in HtrA4-KD cells. For each, the dot plot displays the top 15 ranked GO and KEGG terms, with gene counts indicated by dot sizes and significance by ($-\log_{10}$[P-value], the larger the more significant) on the X-axis.
